# C-Jun N-Terminal Kinase (JNK) Mediates Wnt5a-Induced Cell Motility Dependent or Independent of RhoA Pathway in Human Dental Papilla Cells

**DOI:** 10.1371/journal.pone.0069440

**Published:** 2013-07-03

**Authors:** Chenglin Wang, Yuan Zhao, Yingying Su, Ruimin Li, Yunfeng Lin, Xuedong Zhou, Ling Ye

**Affiliations:** 1 State Key Laboratory of Oral Diseases, Sichuan University, Chengdu, Sichuan, China; 2 West China School of Stomatology, Sichuan University, Chengdu, Sichuan, China; University of California, San Diego, United States of America

## Abstract

Wnt5a plays an essential role in tissue development by regulating cell migration, though the molecular mechanisms are still not fully understood. Our study investigated the pathways involved in Wnt5a-dependent cell motility during the formation of dentin and pulp. Over-expression of Wnt5a promoted cell adhesion and formation of focal adhesion complexes (FACs) in human dental papilla cells (hDPCs), while inhibiting cell migration. Instead of activating the canonical Wnt signal pathway in hDPCs, Wnt5a stimulation induced activation of the JNK signal in a RhoA-dependent or independent manner. Inhibiting JNK abrogated Wnt5a-induced FACs formation but not cytoskeletal rearrangement. Both dominant negative RhoA (RhoA T19N) and constitutively active RhoA mutants (RhoA Q63L) blocked the Wnt5a-dependent changes in hDPCs adhesion, migration and cytoskeletal rearrangement here too, with the exception of the formation of FACs. Taken together, our study suggested that RhoA and JNK signaling have roles in mediating Wnt5a-dependent adhesion and migration in hDPCs, and the Wnt5a/JNK pathway acts both dependently and independently of the RhoA pathway.

## Introduction

Cell migration is a highly integrated multi-step process that orchestrates morphogenesis throughout embryonic development. During gastrulation, large groups of cells migrate collectively as a sheet to form the resulting three-layer embryo. Subsequently, cells migrate from various embryonic layers to their target locations, where they differentiate into the specialized cell types that make up different tissues and organs [1–3]. Analogous migrations occur in tooth development, dental papilla cells migrate and relocate to the enamel-dentinal junction, and those adjacent to dental epithelial cells begin to differentiate into pre-odontoblasts, responsible for dentin matrix secretion and mineralization. Migration and adherence of dental papilla cells to the enamel-dentinal membrane is an essential step in tooth development.

Mammalian tooth development includes different morphological stages, starting with the lamina, bud, cap, and the bell stages, followed by dentin and enamel formation, root formation and tooth eruption [4]. During the progression of dentin formation, dental papilla cells gradually migrate and adhere to the basement membrane (both enamel and dentin form on the relative side of the basement membrane) and differentiate into pre-odontoblasts which are polarized cells [4]. During this complex process, several growth factor families, including Bmp, Fgf, Hh and Wnt, play pivotal roles in mediating tissue formation [5–7].

Wnts participate in a variety of developmental processes during embryonic development in an autocrine or paracrine manner, such as cell proliferation, differentiation, polarity, and migration [8,9]. Secreted Wnts bind to the cell surface and extracellular matrix (ECM), activating either the β-catenin-dependent canonical pathway or β-catenin-independent noncanonical pathway through both the Frizzled trans-membrane receptors (Fz) and the low-density lipoprotein receptor-related protein (LRP) 5/6 co-receptors [10,11]. Wnt4, Wnt5a and Wnt11 are classified as noncanonical Wnt family members and signal *via* noncanonical pathways [12], including the WNT/planar cell polarity (PCP) pathway and the WNT/Ca^2+^ pathway [13,14]. The WNT/PCP pathway controls tissue polarity and cell movement partly through the activation of RhoA and Jun N-terminal kinase (JNK) signaling cascades [15,16].

Wnt5a, a member of the noncanonical Wnt proteins, activates a distinct signal cascade with crosstalk to the canonical Wnt pathway, depending on the receptor context [11,17,18]; e.g. Wnt5a transduces signals through the Frizzled, Ror1, Ror2 or RYK receptors to β-catenin-TCF/LEF, DVL-RhoA-ROCK or DVL-Rac-JNK signaling cascades in a context-dependent manner [19]. The RhoA signaling cascade induces actin cytoskeletal re-organization and cell movement [20]. JNK is activated by Wnt5a and mediates the action of Wnt5a to control convergent extension (CE) movement in *Xenopus* [21]. RhoA activates JNK, which is downstream of the PCP pathway during CE movement in *Xenopus* , and loss of RhoA can be rescued by over-expression of JNK1 [22]. It has been shown that Wnt5a can stimulate migration and invasion in some cell types [23–26], while inhibiting proliferation, migration and invasiveness in others [27,28], which strongly suggests a cell-type-specific effect, along with differential signal transduction.

Previous studies of the gene expression profiles of tooth germ or dental papilla cells indicated that wnt5a mRNA was strongly expressed in murine dental papilla mesenchyme from the bud stage to the bell stage, especially in differentiating odontoblasts [29,30]. Our previous study also found that Wnt5a protein was expressed in odontoblast layers and dental papilla tissues from the early bell stage to the dentin formation stage of human tooth development [31], suggesting that over-expression of Wnt5a could promote differentiation of human dental papilla cells (hDPCs) [32]. A recent study showed that Wnt5a-deficient mice exhibited retarded tooth development with delayed odontoblast differentiation at the early bell stage, leading to formation of smaller and abnormally patterned teeth with delayed odontoblast differentiation at birth [30]. These studies suggested that Wnt5a might play a role in regulating the differentiation processes from dental papilla cells to odontoblasts, although the underlying mechanism of Wnt5a regulation of the adhesion and migration of hDPCs remains unknown.

## Materials and Methods

### Cell Culture, Transfection, and Conditioned Medium Preparation

This study was approved by the Ethics Committee of State Key Laboratory of Oral Diseases of Sichuan University. All study participants gave written informed consents and the samples were obtained from aborted fetuses from West China Women’s and Children’s Hospital of Sichuan University. The dental papilla tissue was isolated from 20-week-old embryos, and human dental papilla cells were cultured following digestion with type I collagenase (2mg/ml) for approximately 45 min, and recombinant adenovirus construction and transfection proceeded as previously described [33,34]. Experiments were carried out using the third and fourth generation of hDPCs. Additional adenoviruses were produced in the same way to express RhoA T19N, RhoA Q63L, or WT RhoA (pcDNA3-EGFP-RhoA-T19N cDNA was kindly provided by Prof. Carolyn Gibson, and pcDNA3-EGFP-RhoA-WT and pcDNA3-EGFP-Q63L cDNA were ordered from www.addgene.org). Wnt5a conditioned medium (CM) or GFP CM were harvested from a confluent monolayer of hDPCs that had been infected with Ad-Wnt5a [34] or Ad-GFP and grown in Dulbecco’s modified Eagles medium (DMEM, Gibco-BRL Life Technologies, Grand Island, NY) containing 10% fetal bovine serum (FBS, Hyclone, South Logan, UT) for 24 hr and subsequently incubated for 48 hr in serum-free DMEM. Typically, CM is stored at -80°C after being centrifuged at 2000 rpm for 5 min and filtered through a 0.22 µm filter. Once thawed, medium was kept refrigerated and retained activity for several weeks [35].

### Cell Adhesion Assay

The cell adhesion assay was performed as previously described [36]. HDPCs were trypsinized, counted using a hemocytometer, and then seeded into 96-well plates coated with type I collagen from rat tail (Sigma-Aldrich, SL, USA) at a concentration of 2.5×10^4^ cells/well, with 50µl 50ng/ml rhWnt5a (R&D Systems, Inc., Minneapolis, MN, USA) or Wnt5a CM for 5, 15 and 30 min (for SP600125, which is from Chemicon, the cells were pre-incubated with 30 µM SP600125 for 30 min at room temperature before being seeded onto a 96-well plate). At each time point, the incubation was stopped by aspirating the floated cells, rinsing the well with 1×PBS, fixing the cells with 4% paraformaldehyde (PFA) and staining the cells with 0.1% crystal violet. Cell density was determined spectrophotometrically by dissolving the stain in the fixed cells with 10% acetic acid and measuring absorbance at OD 570nm. Each time point was assayed in triplicate and each experiment was repeated three times.

### Immunofluorescent Staining

For phalloidin staining and vinculin immunostaining, hDPCs were seeded on glass coverslips coated with type I collagen from rat tail in 50ng/ml rhWnt5a or Wnt5a CM for 15 min (for SP600125, the cells were pre-incubated with 30 µM SP600125 for 30 min at room temperature before seeding on coverslips, and the followed medium also contained 30µM SP600125). For β-catenin immunostaining, hDPCs were grown on glass coverslips to 50~80% confluence and then cultured in 50ng/ml rhWnt5a or Wnt5a CM for 1 hr. Then the hDPCs were fixed with 4% PFA for 15 min and permeabilized with 0.1% Triton X-100 in 1×PBS for 5 min. After blocking with 1% BSA-4% goat serum in PBS for 30 min at room temperature, the cells were incubated at room temperature with either mouse anti-vinculin (1:400 dilution, Sigma-Aldrich, SL, USA) or rabbit anti-β-catenin (1:1000 dilution, Cell Signaling Technology Inc., MA, USA) as primary antibody in 1% BSA with 1×PBS, followed by fluorescent labeled goat anti-mouse or goat anti-rabbit Alexa Fluor 488 or 546 (1:1000; Invitrogen) for 60 min at room temperature. Cells were then washed, mounted in anti-fade reagent and fluorescence microscopy images were taken using an Axioplan Epifluorescence microscope (Carl Zeiss, Jena, Germany) with 20× or 40× objective lens. The number of FACs in at least 100 cells was counted and statistical analysis, and the frequency of different number of FACs was analyzed too. For analysis of cytoskeleton rearrangement, the gray analysis of the fluorescence of F-actin excluding the range of cell nucleus which is highlighted, and the relative fluorescence were analyzed statistically.

### Wound Healing Assay

To carry out the wound-healing assay, the cells were plated onto 6-well-plates coated with 10µg/ml type I collagen from rat tail. The mono-layer of hDPCs was scratched manually with a yellow plastic pipette tip and washed with PBS. The wounded monolayer of cells was allowed to heal for 10-20 hr in 50ng/ml rhWnt5a or Wnt5a CM containing 5% FBS (for SP600125, the cells were pre-incubated with 30 µM SP600125 for 30 min at room temperature before being scratched across the well, and new 30 µM SP600125 was added into the cultured CM). An inverted microscope (Diaphot, Nikon Corporation, Tokyo, Japan) was used to obtain wound healing images. Relative rates of wound closure were measured and expressed as a percentage of the initial length at zero time, with rhWnt5a or Wnt5a CM compared to control medium. Each experiment was repeated three times.

### Western Blot Analysis

HDPCs were grown to 90% confluence followed by serum starvation for 2 hr, and then were treated with 50ng/ml rhWnt5a or Wnt5a CM for different times from 5 to 120 min. Cell lysates (60 µg of protein/lane) were subjected to electrophoresis in 6-12% SDS-PAGE gels. The resolved proteins were transferred electrophoretically to PVDF membrane blots. The blots were incubated with primary antibodies as following: anti-RhoA, anti-phospho-JNK (Tyr183/Tyr185), anti-phospho-MLC (Thr18/Ser19), anti-phospho-paxillin (Tyr118), anti-GAPDH (Cell Signaling Technology Inc., MA, USA) are all diluted 1:1000 overnight at 4°C and HRP-conjugated secondary antibodies (Cell Signaling Technology, 1:1000) for 1 hr at room temperature.

For β-catenin analysis, hDPCs were cultured with Wnt5a CM for 1 hr and then cytoplasm-cell-lysate and nuclei-cell-lysate were obtained following the manufacturer’s protocol with ProteoJet cytoplasmic and nuclear protein extraction kit (Fermentas, Glen Burnie, MD, USA). Primary antibodies (anti-β-catenin, anti-H3) were from Cell Signaling Technology Inc.

### RhoA Pull-down Assay

Pull-down assay with a glutathione transferase (GST) fusion protein containing the RhoA binding domain of rhotekin (rhotekin-GST) was performed essentially as described in the manufacturer’s protocol for GTPase Pull-Down kit (Thermo Scientific, Waltham, MA, USA). Samples were analyzed for activated and total RhoA by Western blot analysis using anti-RhoA antibody.

### Statistical Methods

**Figure 1:**
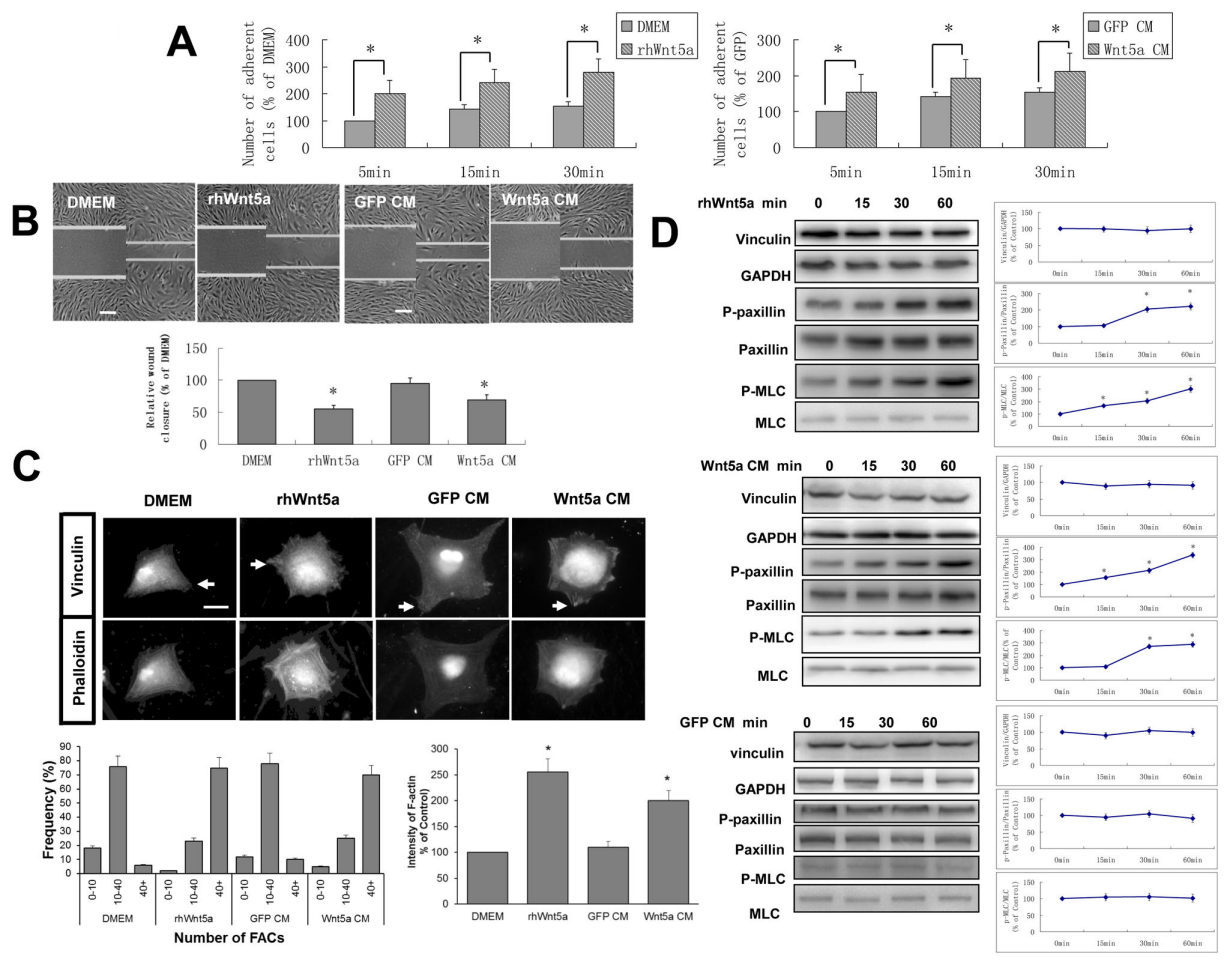
Wnt5a promotes the adhesion but inhibits the migration of hDPCs. A: A total of 25,000 cells were seeded for the indicated times and nonadherent cells were rinsed off. Adherent cells were stained with crystal violet and were analyzed spectrophotometrically. The number of adherent cells is shown as mean±SD from three independent experiments, with the number of DMEM-treated cells set as 100%. B: Confluent monolayers of hDPCs were scratched with a pipette tip and cultured in different medium containing 5% FBS for 12 hr. The relative migration distance of the wound edge was shown as mean±SD of three independent experiments, with the migration distance of DMEM-treated cells set as 100%. Bars, 100 µm. C: HDPCs were plated on glass coverslips coated with type I collagen and cultured with different medium for the indicated times. For FACs immunostaining, anti-vinculin antibody was used, and for F-actin staining, rhodamine-phalloidin was used, arrowheads mark FACs. Bars,10 µm. D: Confluent hDPCs were incubated with Wnt5a for the indicated times and Western analyses were used to detect the expression of vinculin, phospho-paxillin, phospho-MLC, with GAPDH, total-paxillin and total-MLC as loading control. The relative protein expression at 0 min is designated 1.0. *p < 0.05, n=3.

**Figure 2:**
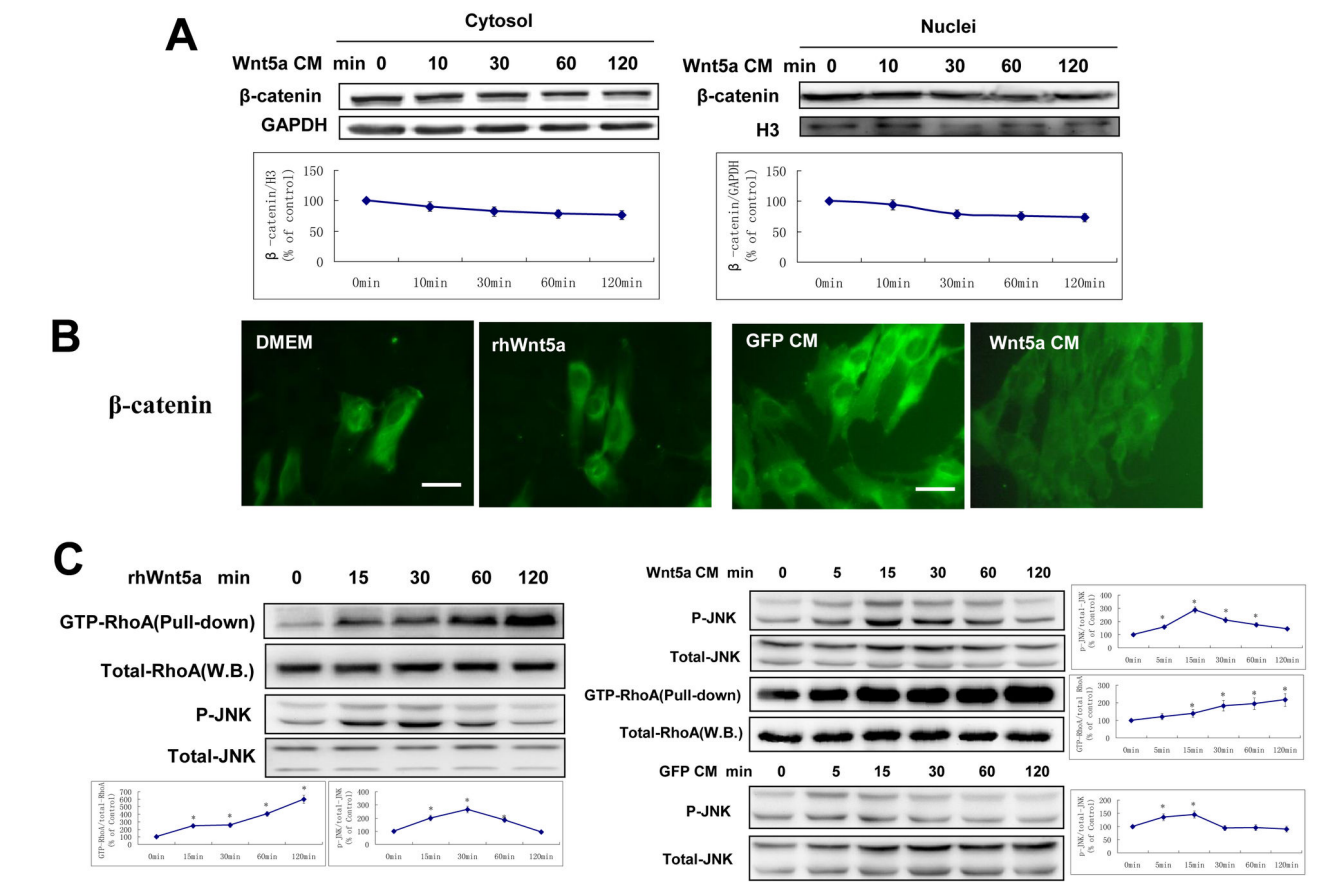
Wnt5a has no effect on β-catenin expression or translocation in hDPCs. A: Western analyses of β-catenin in cytosolic or nuclear fractions of cells cultured in Wnt5a CM for indicated times. Cytosolic and nuclear signals were normalized to GAPDH and H3, respectively. The relative expression of β-catenin at 0min is designated 1.0. B: Immunofluorescence microscopy of hDPCs following culture with rhWnt5a or Wnt5a CM for 1 hr. β-catenin signal is in green. Bars,30 µm. C: Wnt5a up-regulates the expression of GTP-RhoA and phospho-JNK. RhoA activity stimulated by Wnt5a was detected by GST-Pull down assay at the indicated times, the expression of total RhoA, phospho-JNK, and total-JNK was detected by Western blot analysis. The relative protein expression at 0min is designated 1.0. *p < 0.05, n=3.

**Figure 3:**
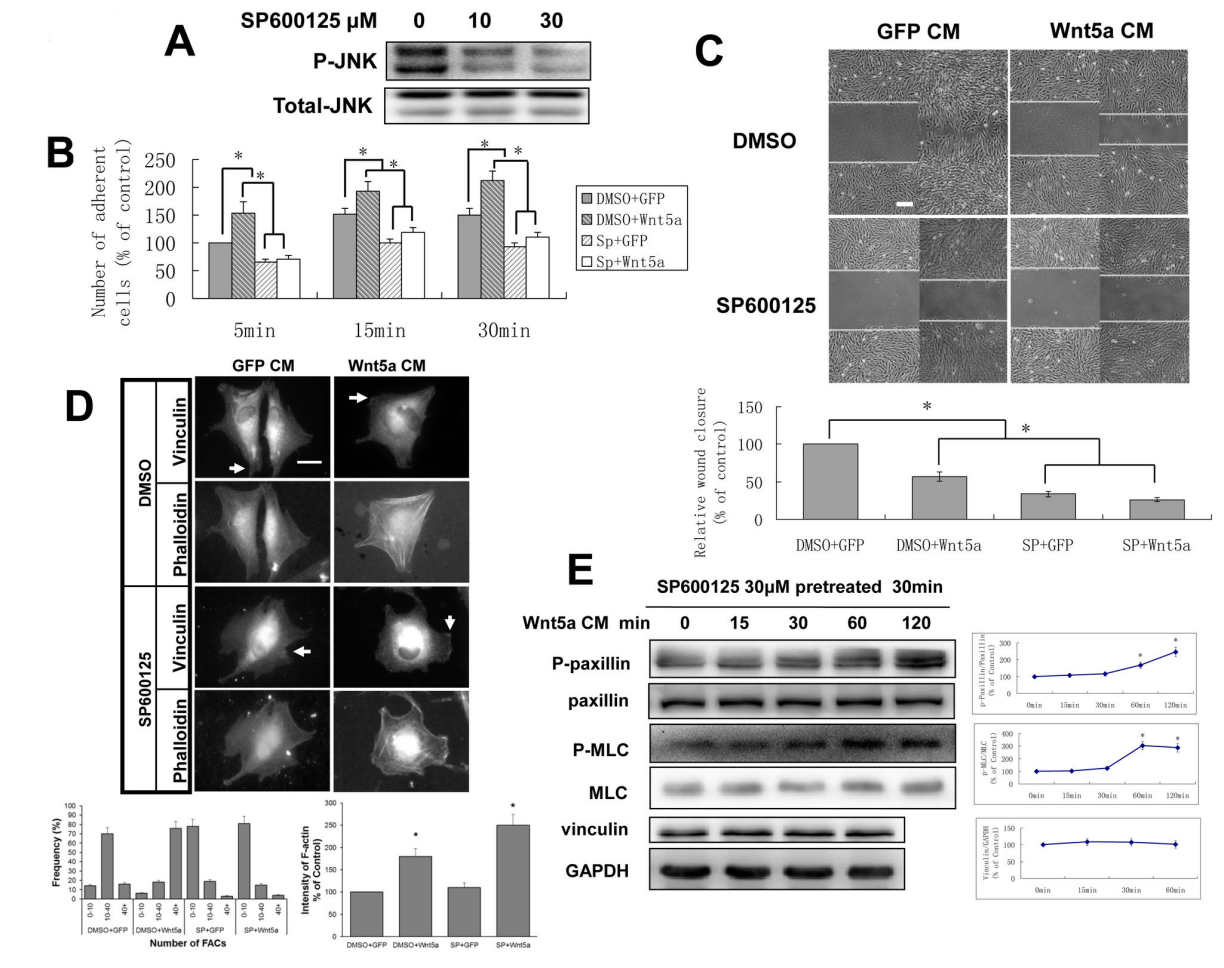
The role of the JNK pathway in Wnt5a-dependent adhesion, migration and formation of FACs. A: HDPCs were pretreated with 10 µM or 30 µM SP600125 for 30 min and the levels of phospho-JNK and total JNK were measured by Western blot analysis. The relative protein expression without SP600125 treatment is designated 1.0. B ,C: Cell adhesion and wound healing assays were performed as in Figure 1, but the cells were hDPCs which were pretreated with 30 µm SP600125 for 30 min, and the observed time in the wound healing assay was 16 hr. Bars, 100 µm. D: Vinculin immunostaining and phalloidin staining were performed at 15min as in Figure 1C, but the hDPCs were pretreated with 30µM SP600125 for 30 min before being seeded onto glass slides. Arrowheads mark FACs. The number of FACs and the relative fluorescence were analyzed as in Figure 1C. Bars,10 µm. E: Pretreatment with 30 µM SP600125 for 30 min, hDPCs were incubated with Wnt5a CM for the indicated times, the cell lysates were collected and immunoblotted with antibodies to phospho-MLC, phospho-paxillin, total-paxillin, total-MLC and vinculin. The promotion of phospho-paxillin by Wnt5a CM was delayed until after 60 min, but no changes were seen in the expression of phospho-MLC. *p < 0.05, n = 3.

**Figure 4:**
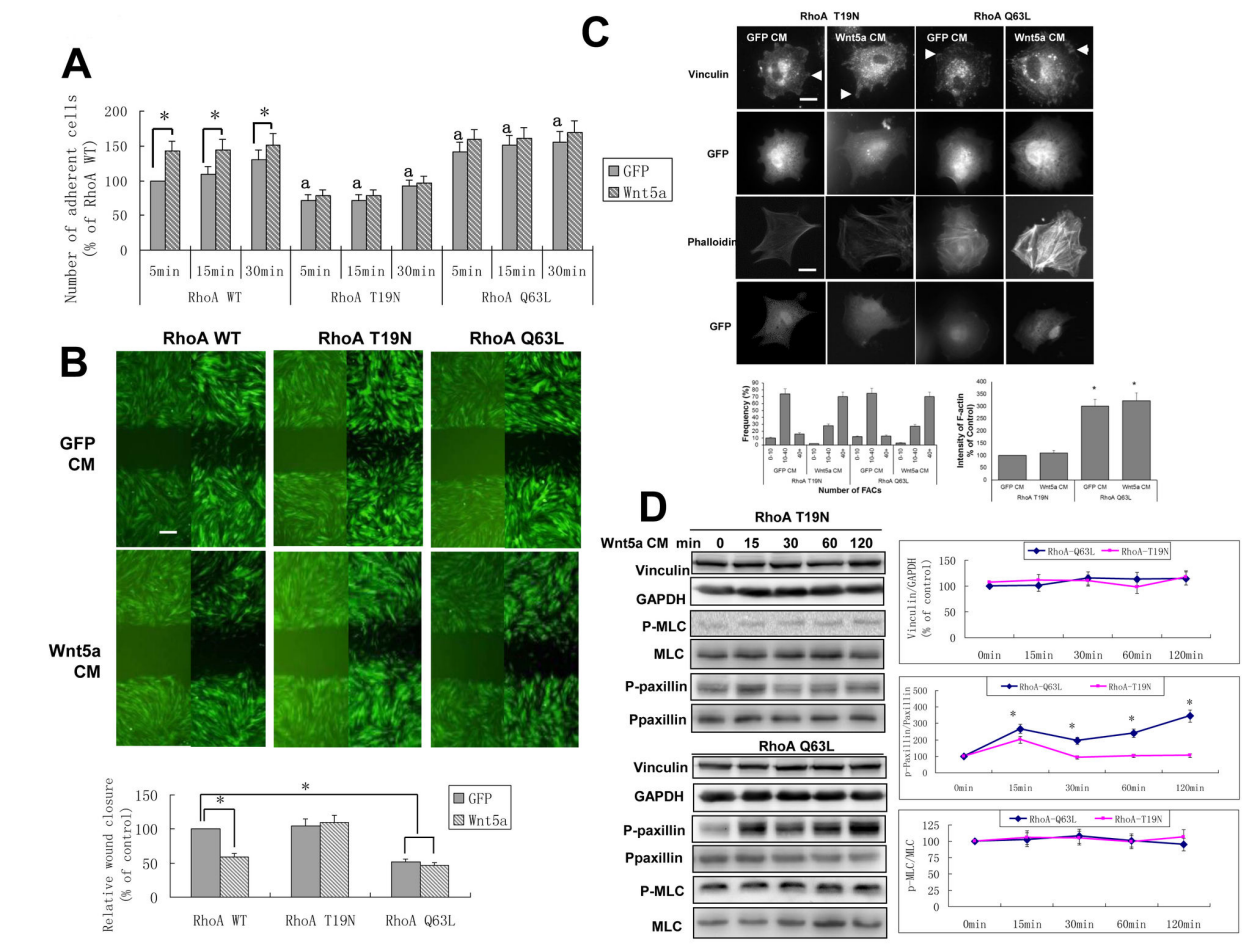
RhoA signaling contributed to the Wnt5a-dependent adhesion, migration changes and formation of FACs. A: After infection with RhoA mutant adenoviruses, hDPCs were cultured with GFP CM or Wnt5a CM and cell adhesion assays were performed, as in Figure 1A. B: Confluent hDPCs infected with RhoA mutant adenoviruses for 48 hr were scratched and cultured with GFP CM or Wnt5a CM for 20 hr to observe the effect of Wnt5a CM on the cell. Bars,100 µm. C: Vinculin immunostaining and phalloidin staining were performed as in Figure 1C, but the hDPCs were infected with different RhoA mutant adenoviruses and the secondary antibody was Alexa 546-labeled. Arrowheads mark FACs. The number of FACs and the relative fluorescence were analyzed as if Figure 1C. Bars, 10 µm. D: After infection with RhoA T19N or RhoA Q63L adenoviruses for 48 hr, hDPCs were incubated with Wnt5a CM for the indicated times and collected for protein extraction. Western analyses of phospho-MLC, phospho-paxillin, total-paxillin, total-MLC and vinculin in hDPCs. a *p* < 0.05, n = 3, compared with RhoA WT at the same time point. *p < 0.05, n = 3.

**Figure 5:**
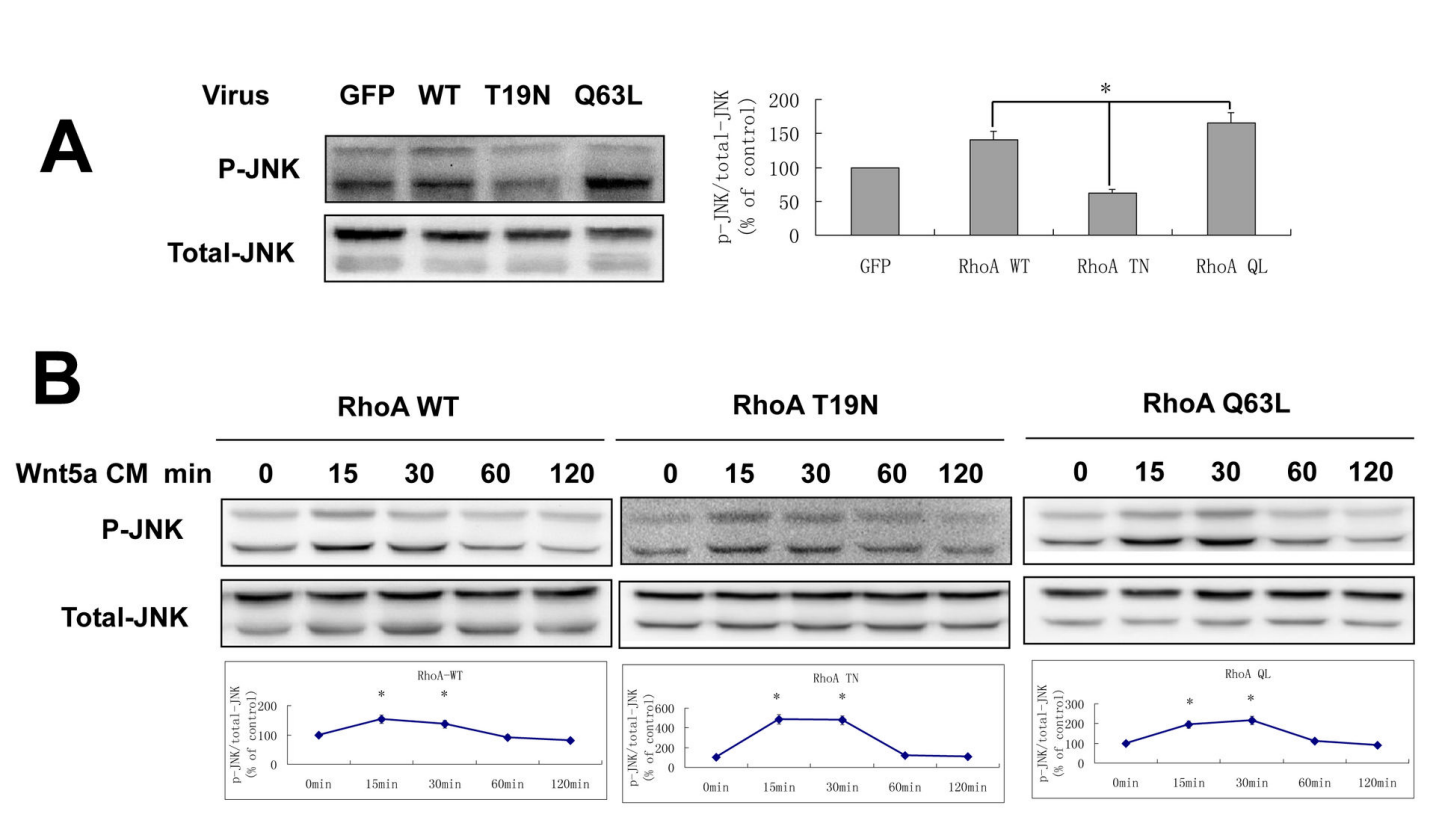
Wnt5a activated JNK signaling is dependent and independent of RhoA signaling. A: Lysates from hDPCs were obtained following transfection with adenoviral vectors encoding GFP, RhoA WT, RhoA T19N and RhoA Q63L for 48 hr and the levels of phospho-JNK and total-JNK were measured by Western blot analysis. The normalized amount of phospho-JNK in GFP adenovirus infected hDPCs is designated 1.0. B: HDPCs infected with adenoviruses encoding the RhoA mutant for 48 hr were cultured with Wnt5a CM and the cell lysates were obtained at the indicated times, with the level of phospho-JNK and total-JNK measured by Western analyses. The relative amount of phospho-JNK is normalized to the total amount, at 0min, is designated 1.0. *p < 0.05, n=3.

Statistical analyses for Figures 1-5 were carried out using SPSS13.0 software; Student’s t-test was applied. *P*-value less than 0.05 were considered statistically significant.

## Results

### Wnt5a increased the adhesion of hDPCs, while decreasing migration

HDPCs were derived from tooth germs and cultured as previously described [33]. Wnt5a CM was obtained from hDPCs transfected with adenoviral vectors encoding the wnt5a gene [34]. GFP (green fluorescent protein) CM was prepared from hDPCs transfected with control adenoviral vectors which carry the gene encoding GFP.

In order to test the effect of exogenous Wnt5a on cell adhesion to the ECM, cell adhesion assays were performed. When plated to type I collagen-coated wells, hDPCs with rhWnt5a or Wnt5a CM showed greater adhesion than hDPCs with control medium or GFP CM at 5, 15, 30 min (Figure 1A).

Based on the effect of Wnt5a on cell-ECM adhesion of hDPCs, we further investigated the influence of Wnt5a on the migration of hDPCs using a wound healing assay [24] and found that Wnt5a inhibited the migration of hDPCs (Figure 1B). The results were consistent with our previous study of endogenous Wnt5a protein with wound healing assays [34] and suggest that exogenous Wnt5a has a similar effect on hDPCs.

### Wnt5a promoted the formation of focal adhesion complexes and the rearrangement of cytoskeleton, up-regulated the phosphorylation of myosin light chain and paxillin

In fibroblasts, focal adhesion complexes (FACs) can be observed at the leading edge and attach to the ECM during the process of cell adhesion and migration [1]. FACs are mostly composed of β1, β3 integrins and some structural proteins including talin, vinculin, paxillin, et al. [37]. RhWnt5a or Wnt5a CM stimulation significantly enhanced the formation of FACs along the rearranged cytoskeleton, with more FACs formation at 15 min (Figure 1C), while not changing the expression of vinculin in hDPCs (Figure 1D). The results suggested that some signal pathways activated by Wnt5a could promote the formation of FACs at the early stage of cell movement.

Paxillin, an integrin assembly adaptor protein, can be recruited to the leading cell edge promptly upon the initiation of migration and integrates diverse signals from tyrosine kinase and Rho family GTPase [38,39]. Paxillin has four major tyrosine phosphorylation sites with the phosphorylation of Tyr31 and Tyr118 highly augmented during cell adhesion and migration and present at the leading edges of migratory cells [39–42]. By Western blot analysis, we found that, consistent with the promotion of the FACs formation, Wnt5a up-regulated the expression of phospho-paxillin at Tyr118 sites at 15 min (Figure 1D). Myosin light chain (MLC) 2 is phosphorylated at Thr18 and Ser19 by myosin light kinase, and ROCK can also phosphorylate Ser19 of MLC2, which regulates the assembly of stress fibers. Our study shows that Wnt5a up-regulated the expression of F-actin and phospho-MLC at the Ser19 site at 30min (Figure 1C, D). Both results suggest that the Wnt5a-promoted cell adhesion was correlated with the formation of FACs and the phosphorylation of paxillin.

### Wnt5a had no effect on β-catenin expression or translocation but activated RhoA and JNK signaling in hDPCs

β-catenin is known to interact with E-cadherin [43,44], a cell-cell adhesion molecule, and it has been reported that Wnt5a could promote the formation of β-catenin/E-cadherin complexes on the cell membrane, promoting cell-cell adhesion and inhibiting cell migration in human breast epithelial cells [45]. Based on the observation that Wnt5a inhibited monolayer cell migration of hDPCs, we first examined the effect of Wnt5a on β-catenin in our cells.

Although Wnt5a did activate canonical Wnt/β-catenin signaling in mammalian cells while over-expressing Fz4 [18], Wnt5a failed to activate either expression of β-catenin or its translocation into the nucleus in hDPCs, even showing slight inhibition (Figure 2A). In our study, rhWnt5a or Wnt5a CM did not stimulate nuclear translocation of β-catenin, and β-catenin was localized to the cytoplasm, periplasmic membrane and cell-cell junctions (Figure 2B). These results suggested that Wnt5a did not induce the accumulation of the three different pools of β-catenin, including membrane bound, cytoplasm and nuclear in hDPCs.

In the noncanonical WNT pathway, RhoA or JNK signaling are hypothesized to be involved in the WNT/PCP pathway and regulate cell motility [2,46]. We found Wnt5a up-regulated the phosphorylation of JNK at 15 min and 30 min, and increased RhoA activity in a time-dependent manner from 15 min to 120 min (Figure 2C), while GFP CM had no significant effect (Figure 2C). The activity of RhoA is consistent with the phosphorylation of MLC (Figure 1D), as RhoA/ROCK can phosphorylate Ser19 of MLC2 and promote the assembly of stress fibers.

### Disruption of the JNK pathway resulted in an inhibition of Wnt5a-dependent adhesion, migration and formation of FACs

The JNK cascade participates in the WNT/PCP pathway and WNT/JNK signaling is thought to be involved in controlling CE movement and regulating cell motility [2,46], so we first examined the effect of JNK signaling on Wnt5a-induced motility changes in hDPCs.

Pre-treatment with SP600125, a specific inhibitor of the JNK pathway, blocked the activation of JNK signaling with phospho-JNK reduced ~70% (Figure 3A) and decreased hDPCs adhesion and migration (Figure 3B, C). The effect of Wnt5a CM on hDPCs adhesion has been mostly blocked by SP600125 treatment, and the inhibitory effect of Wnt5a CM on hDPCs migration was further enhanced by treatment with SP600125 (Figure 3B, C). Immunofluorescence of vinculin and phalloidin staining showed that JNK pathway blockade could decrease the formation of FACs but had no effect on the rearrangement of cytoskeleton, and that Wnt5a CM couldn’t rescue FACs inhibition at the early stage of cell movement (15 min) (Figure 3D). Interestingly, Wnt5a CM stimulation still promoted the rearrangement of cytoskeleton when the JNK pathway was blocked (Figure 3D). These results suggested that JNK signaling plays a key role in the cell adhesion of hDPCs and closely relates to Wnt5a-dependent formation of FACs in the early stage of cell movement.

In order to study the regulatory mechanism of Wnt5a on hDPCs when the JNK pathway was blocked, the phosphorylation of paxillin and MLC were tested in hDPCs with SP600125 pretreatment and Wnt5a CM stimulation. We found that the effect of Wnt5a CM on phospho-paxillin was delayed rather than reduced by SP600125 relative to Figure 1D, and JNK pathway blockade had no effect on the phosphorylation of MLC (Figure 3E). These data suggested that Wnt5a-dependent paxillin phosphorylated at Tyr118 was directly and indirectly downstream of JNK signaling in hDPCs, which is different from previous reports stating phosphorylated paxillin was the simple target of JNK signaling [47], as the paxillin was phosphorylated at Ser178.

### RhoA GTPase contributes to the Wnt5a-dependent adhesion and migration changes in hDPCs

As Wnt5a CM stimulation still promotes the rearrangement of cytoskeleton and the phosphorylation of MLC when the JNK pathway was blocked, we further examined the effect of Wnt5a on RhoA signaling in hDPCs.

To address the potential role of RhoA on hDPC cell adhesion and migration, we first constructed replication-deficient recombinant adenoviruses carrying expression plasmids encoding RhoA T19N to express dominant negative RhoA and RhoA Q63L to express constitutively activated RhoA in hDPCs, while wild type (WT) RhoA was used as control [48]. Then, we examined the effect of RhoA mutants on the adhesion and migration of hDPCs, and found that expression of RhoA T19N resulted in decreased cell adhesion but increased cell migration, while RhoA Q63L increased cell adhesion and decreased cell migration (Figure 4A, B). Infection of hDPCs with both RhoA T19N and RhoA Q63L adenovirus for 48 hr blocked the effect of Wnt5a CM on adhesion and migration, while RhoA Q63L showed a similar inhibition of cell migration with or without Wnt5a (Figure 4A, B). These results suggested that RhoA activation plays a key role in Wnt5a-dependent hDPC motility.

Although RhoA T19N and Q63L blocked the effect of Wnt5a CM on the rearrangement of cytoskeleton (Figure 4C), neither RhoA T19N nor Q63L could block Wnt5a CM’s promotion of FACs formation at 15 min (Figure 4C), despite the fact that RhoA can regulate the formation of FACs in different types of fibroblasts [49]. Further study showed that Wnt5a CM promoted the phosphorylation of paxillin at 15 min, regardless of RhoA pathway’s blockade by RhoA T19N or activation by RhoA Q63L (Figure 4D), which corresponds with the effect of Wnt5a CM on the formation of FACs. RhoA T19N or RhoA Q63L inhibited or increased the phosphorylation of MLC, as shown in Figure 4D, contrasting with the expression of phospho-MLC in Figure 1D. After infection with RhoA T19N or RhoA Q63L adenovirus for 48 hr, Wnt5a CM did not up-regulate the expression of phospho-MLC (Figure 4D), which is consistent with the effect on cytoskeleton rearrangement. These data suggested that the phosphorylation of MLC is closely correlated with the activity of RhoA and that Wnt5a can activate MLC through RhoA signaling. This suggested that the Wnt5a-induced formation of FACs and phosphorylation of paxillin in hDPCs have no correlation with RhoA activity or the level of activated RhoA, but Wnt5a-induced rearrangement of cytoskeleton and phosphorylation of MLC have correlation with RhoA activity.

### Wnt5a-JNK signaling mediated hDPCs motility which was dependent and independent of the RhoA pathway

The RhoA/JNK cascade participates in the WNT/PCP pathway to control cell movement, and we found that the activity of JNK is closely related to the activity of RhoA. However, the level of phospho-JNK was altered after treatment with RhoA T19N or RhoA Q63L (Figure 5A), which suggested that JNK could be downstream of RhoA signaling in hDPCs. But hDPCs infected by RhoA mutant adenovirus have no significant changes in the expression of phospho-JNK after stimulation with Wnt5a CM (Figure 5B). These results suggested that Wnt5a could activate the JNK pathway and the process is both dependent and independent of the Wnt5a-RhoA pathway.

## Discussion

Human dental papilla cells, also called human dental papilla mesenchyme cells [50], are the only precursor cells which can differentiate into dental pulp cells and odontoblasts to form a dentin-pulp complex [7,51–53]. Wnt5a is representative of non-canonical Wnts transducing PCP signaling which controls tissue polarity and cell movement through FZD3 or FZD6 receptors and Ror1, Ror2 or PTK7 co-receptors [54]. The dishevelled-dependent WNT/PCP signals are transduced to the RhoA signaling cascade through Formin homology proteins Daam1 and Daam2 and to the JNK signaling cascade through MAPKKKs and MAPKK4/7 [15,55,56]. In this study, we showed that Wnt5a activated the RhoA and JNK signaling cascades to regulate adhesion and migration of hDPCs and that Wnt5a could activate JNK signaling dependent or independent of activated RhoA. This result suggested that RhoA and JNK play different roles in Wnt5a-mediated hDPC motility.

Wnt signaling is receptor context dependent. Wnt5a was shown to activate either the non-cannonical WNT pathway via the PCP and Ca^2+^ pathways [11] or the canonical WNT pathway in the presence of Fz4 and Lrp5 [18]. Wnt5a inhibits canonical signaling by promoting degradation of β-catenin in a GSK-3-independent way [12] or in the presence of Ror2 [18,57]. Considering β-catenin is a multi-functional molecule involved in cell-cell adhesion and signaling, our study first examined the effect of Wnt5a on β-catenin stabilization in hDPCs. The spatiotemporal change of β-catenin mRNA expression in dental papilla was reported in cells which differentiated into odontoblasts [58]. Early studies found that Wnt5a stimulation of human breast epithelial cells leads to increased Ca^2+^-dependent cell-cell adhesion and increased complex formation of β-catenin/E-cadherin [45]. In this study, we showed that Wnt5a had no significantly effect on β-catenin stabilization and nucleus translocation.

In embryonic development, as neural crest cells migrate to the skin, they express high levels of Wnt5a, which results in increased morphogenetic movement in developing cells. When the cells reach their site of differentiation and become melanocytes, the expression of the Wnt5a mRNA drops to very low levels [59]. At present, the studies on Wnt5a in cell migration mostly focused on tumor cells. It has been shown that Wnt5a stimulates migration and invasiveness in some cancer cells like melanoma, breast cancer, lung cancer and gastric cancer [23–25,60,61]. Other studies reported that Wnt5a had the ability to inhibit proliferation, migration and invasiveness in thyroid tumors and colorectal cancer cell lines [27,28]. Our study showed that exogenous Wnt5a protein significantly correlated with inhibition of cell migration and increased cell adhesion. However, the underlying mechanism of how Wnt5a affects cell motility remains unclear.

Previous studies showed that RhoA was strongly expressed during tooth morphogenesis and was present in ameloblasts and odontoblasts during cyto-differentiation [62]. RhoA transmits multiple extracellular signals into intracellular events and ultimately controls cell morphology and a variety of functions, such as cell motility, aggregation, polarity and contraction [63,64]. Even endogenously activated RhoA regulated stem cell lineage commitment by regulating cell shape [65]. Here, we have demonstrated that activated RhoA could affect the adhesion and migration of hDPCs and participate in the regulation of Wnt5a-dependent hDPC motility.

In the process of cell migration, RhoA regulates the assembly of actin stress fibers and associated focal adhesions through activation of its downstream effectors mDia and the ROCKI and ROCKII kinases [49,66]. In cell movement, RhoA activity is required to induce actomyosin contractility following the phosphorylation of MLC, driving the translocation of the cell body retraction at the rear [67]. Constitutively activated RhoA may inhibit cell migration by inducing high cell-skeleton contractility which can be seen in fibroblasts and macrophages [68,69], as well as in our hDPCs. On the other hand, RhoA may also negatively influence cell migration by increasing stress fiber-dependent adhesions to the substrate [70]. Tight control of the RhoA activity seems to be required to balance the opposing effects of cell body contraction and adhesion [71], with the specific mechanism controlling RhoA-inhibited cell migration not been well understood [72]. In our study, Wnt5a increased hDPCs adhesion and inhibited hDPCs migration through the RhoA signaling pathway, possibly through promotion of cell contractility and cell adhesion. Interestingly, Wnt5a had a positive effect on hDPCs cytoskeletal contractility through the RhoA signaling pathway with up-regulated expression of phospho-MLC. While having a positive effect on hDPCs adhesion, increasing the formation of FACs and the expression of phospho-paxillin, the specific mechanism of Wnt5a on hDPCs adhesion and migration requires further study.

As a structural protein in focal adhesions, paxillin was involved in the dynamics of the structure and tyrosine phosphorylation is one of the key signaling events occurring at focal adesions [39,73]. A previous study reported that paxillin phosphorylation at Tyr31/118 could suppress RhoA activity and promote efficient membrane spreading and ruffling at the early stage of cell adhesion and migration [41]. In our study, we found that Wnt5a/JNK signaling could phosphorylate paxillin at Tyr118 and promoted the formation of FACs, but the mechanism of phospho-paxillin mediation of RhoA activity in hDPCs still need more research.

The ability of RhoA to stimulate JNK provides a molecular mechanism through which Wnt5a may act, as reported in a variety of cellular systems [63,74,75]. The RhoA/JNK pathway also participates in developmental morphogenetic processes, as suggested by genetic epistasis studies in *Drosophila* indicating that JNK mediates the generation of tissue polarity induced by RhoA [76]. Other reports showed that Wnt5a can activate JNK signaling and that activated JNK will help with accurate CE movements [21], while Ror2 is involved in the non-canonical Wnt5a/JNK signaling pathway [57,77,78]. Some authors have demonstrated that JNK activity plays a critical role in the migration of fibroblasts in wound healing assays using a gene knockout approach [79]. In this study, Wnt5a could activate JNK signaling dependent or independent of activated RhoA, and Wnt5a-dependent JNK signaling activation promotes the formation of FACs, while the expression of phospho-paxillin at Tyr118 is not mediated by the Wnt5a-RhoA signaling pathway.

**Figure 6:**
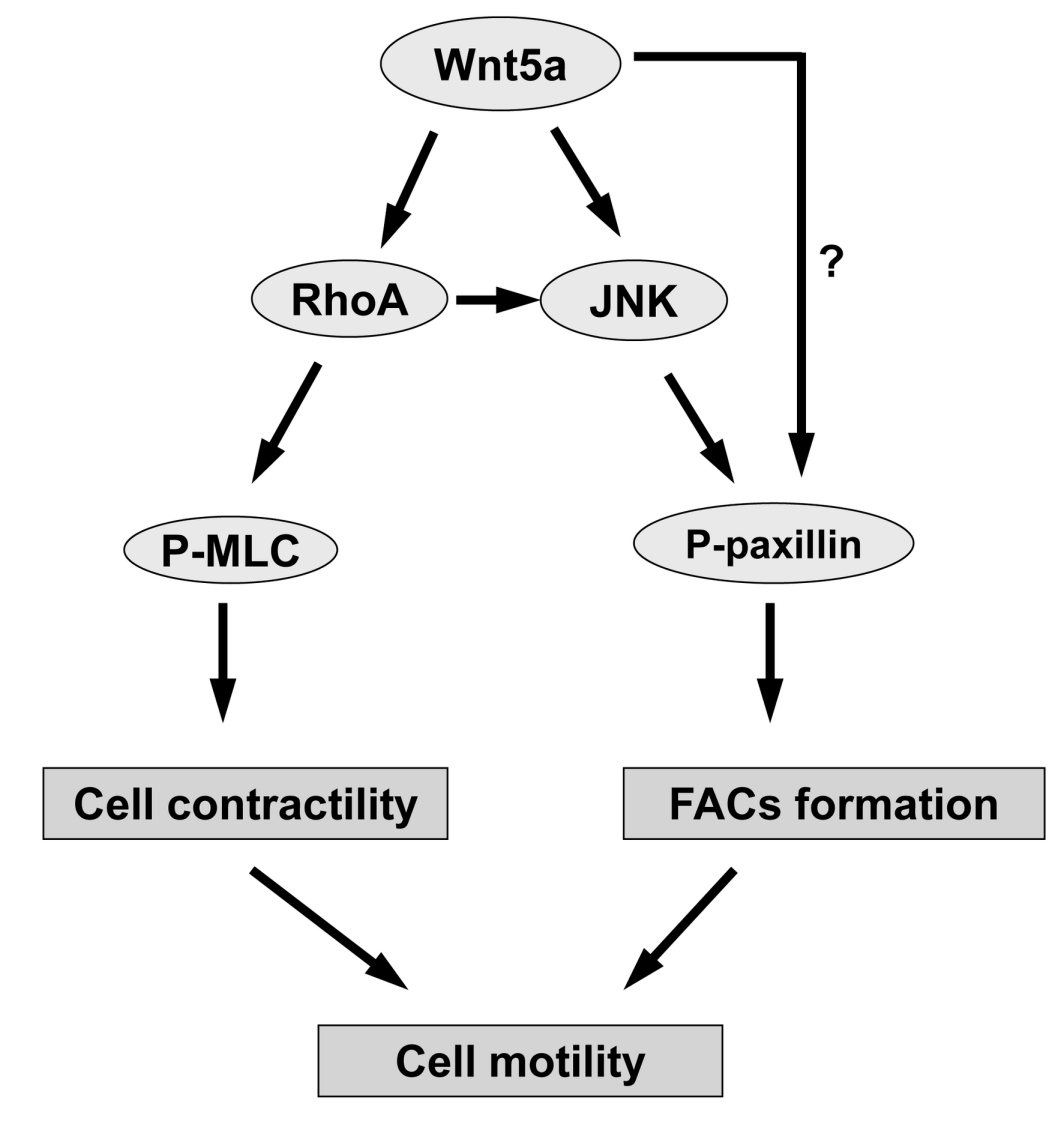
The role of the RhoA and JNK pathways in Wnt5a-dependent hDPC motility.

In summary, Wnt5a activated JNK signaling dependent or independent of the RhoA pathway, which leads to an increased formation of FACs. Tyr31/118-phosphorylated paxillin participated in this process, and perhaps suppresses RhoA activity [41]. Wnt5a activated the RhoA and JNK signaling pathways, and then up-regulated the expression of phospho-MLC for the increase of cytoskeletal rearrangement and Tyr118-phosphorylated paxillin for increased formation of FACs, finally leading to increased cell contractility and adhesion, resulting in inhibition of hDPC migration (Figure 6).

The scheme represents a work-in-progress of our understanding of Wnt5a-stimulated pathways involved in hDPC motility. Wnt5a can activate the RhoA signal and promote the expression of phospho-MLC, which is followed by cell contractility. Meanwhile, Wnt5a can activate JNK signaling dependent and independent of the RhoA pathway, followed by expression of phospho-paxillin and formation of FACs. Both RhoA and JNK signaling regulates the Wnt5a-dependent cell motility of hDPCs.
